# Accounting for Pacific climate variability increases projected global warming

**DOI:** 10.1038/s41558-024-02017-y

**Published:** 2024-06-05

**Authors:** Yongxiao Liang, Nathan P. Gillett, Adam H. Monahan

**Affiliations:** 1 School of Earth and Ocean Sciences, University of Victoria, Victoria, British Columbia Canada; 2 Climate Research Division, Environment and Climate Change Canada, Victoria, British Columbia Canada

**Keywords:** Projection and prediction, Climate and Earth system modelling

## Abstract

Observational constraint methods based on the relationship between the past global warming trend and projected warming across climate models were used to reduce uncertainties in projected warming by the Intergovernmental Panel on Climate Change. Internal climate variability in the eastern tropical Pacific associated with the so-called pattern effect weakens this relationship and has reduced the observed warming trend over recent decades. Here we show that regressing out this variability before applying the observed global mean warming trend as a constraint results in higher and narrower twenty-first century warming ranges than other methods. Whereas the Intergovernmental Panel on Climate Change assessed that warming is unlikely to exceed 2 °C under a low-emissions scenario, our results indicate that warming is likely to exceed 2 °C under the same scenario, and hence, limiting global warming to well below 2 °C will be harder than previously anticipated. However, the reduced uncertainties in these projections could benefit adaptation planning.

## Abstract (web summary)

Internal variability can strongly influence global temperature trends. Here the authors show that if the internal variability in the eastern tropical Pacific is removed from recent trends, the constrained projected warming with future CO_2_ emissions is higher than currently expected.

## Main

Observational constraints (also known as emergent constraints) provide an effective way to narrow the uncertainty in multi-model projections of climate warming under a given emissions scenario^1–10^. Relationships between an observational constraint and projected warming across models result from differences in the representation of key climate response processes between climate models^9–11^. Such a relationship can be used together with observations of the metric concerned to derive observationally constrained projections. An underlying assumption of emergent constraint analyses is that the emergent relation identified in the models is applicable to the real world^11^.

The observed recent trend in global mean near-surface air temperature (GSAT) is a promising constraint for future global mean warming, since it combines the effects of many individual feedbacks controlling the climate response. Since the major feedbacks that control the individual models’ climate response do not change substantially over time, the recent GSAT trend should be highly correlated with future projected warming across a set of climate models^1,2,11^. However, the historical GSAT trend is sensitive to the influence of decadal to multi-decadal internal variability^6,12–15^. The past warming trend is, therefore, not a pure externally forced trend, and the resulting constrained projections could be biased by the strong influence of internal variability in observations^6,14^.

Previous studies have shown that the recent warming trend (particularly since the 1990s) in observations is smaller than that of most Coupled Model Intercomparison Project Phase 6 (CMIP6) climate model simulations^1,16^. This can be partly understood as a product of internal variability driven by the so-called pattern effect (associated with warming in the western equatorial Pacific Ocean and cooling in the eastern tropical Pacific (ETP))^17–19^. A relatively cold sea surface temperature (SST) trend pattern in the ETP results in a substantial negative cloud radiative feedback and, therefore, in a lower global mean warming trend^14,18,20–22^. Removing the effect of such internal variability makes the relatively muted warming observed in the tropical troposphere consistent with simulated warming even in high climate sensitivity models^14^. In this Article, our objective is to enhance the robustness of future warming projections by reducing the influence of internal variability on past warming trends when using them as observational constraints.

## Tropical Pacific SST trends

**Fig. 1:**
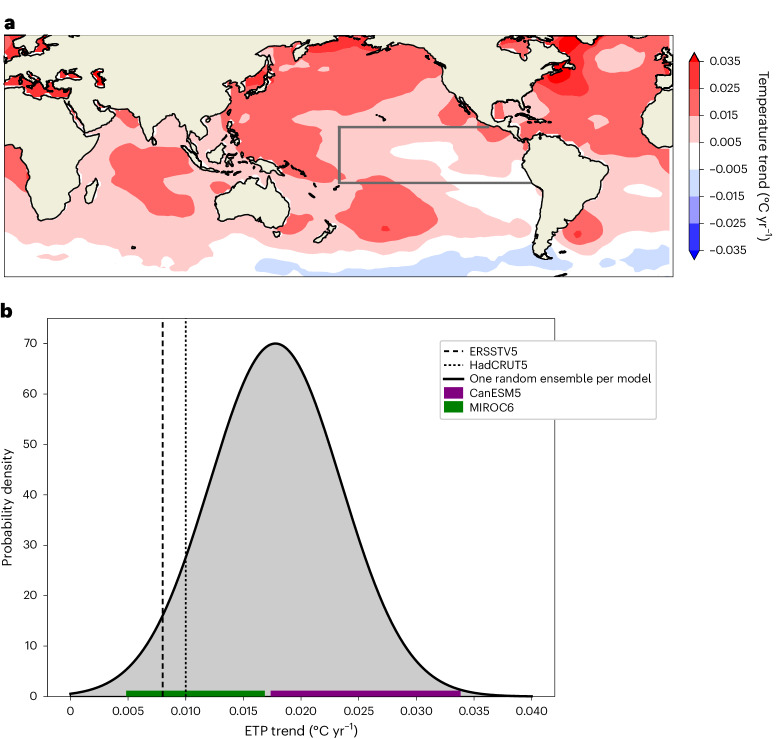
Simulated and observed 1970–2022 SST trends. **a**, SST trends in ERSSTv5 (ref. ^42^) observations. The grey box shows the ETP region. Following Kosaka and Xie^17^, the ETP is defined as the region in the Pacific east of the dateline to the South American coastline between 20° S and 20° N. **b**, The distribution of the ETP SST trend in the CMIP6 multi-model ensemble. The distribution is determined by randomly sampling one realization per model 5,000 times, fitting a Gaussian to the multi-model distribution and averaging these sample distributions. The horizontal bars show the ranges (from minimum to maximum) across 50-member ensembles for individual models CanESM5 and MIROC6. The standard deviations of ETP trends are 0.003 °C yr^−1^ in CanESM5 and MIRCO6 and 0.006 °C yr^−1^ in the full CMIP6 ensemble. The vertical lines represent the ETP trend based on different observation datasets.

Previous studies find that climate models do not on average capture the observed pattern of SST trends (Fig. 1a) over the tropical Pacific well^23,24^. Consistent with this, Fig. 1b shows that over the 53-year period from 1970 to 2022, most coupled model realizations produce a stronger warming trend in the ETP than observed. However, Fig. 1b also shows that the observed ETP SST trend is reproduced in a few individual simulations from the CMIP6 models, consistent with previous studies^19^. Moreover, large ensembles of simulations from individual models (horizontal bars in Fig. 1b), which differ only in their initial conditions, span a substantial fraction of the CMIP6 model spread (black probability density function (PDF)), suggesting that internal variability is an important contributor to the variability of the ETP trend.

Decadal variability over the Pacific Ocean in CMIP6 models shows an improved performance (both for spatial structure and magnitude) relative to models in CMIP5 (ref. ^25^). The fact that the spread of ETP trends in the multi-model CMIP6 ensemble with its increased ensemble sizes encompasses the observations suggests that the observed ETP cooling pattern may be explained as a rare realization of internal variability. The relatively low observed GSAT trend induced by such internal variability will tend to result in relatively low constrained warming projections when applying GSAT trend as a predictor.

**Fig. 2:**
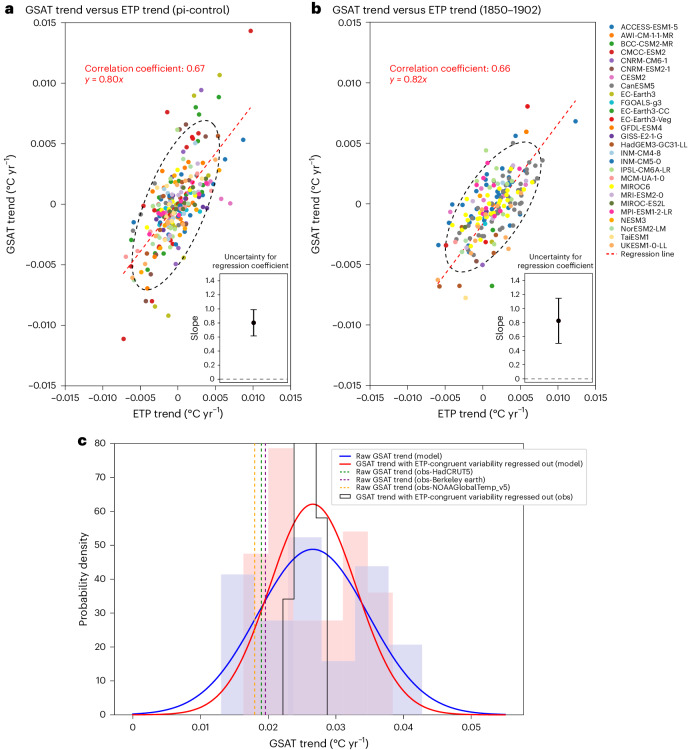
Influence of unforced ETP internal variability. **a**, Scatter plot showing the relationship between the GSAT trend and ETP trend from pre-industrial control simulations. The last 477 years of each control simulation was divided into nine 53-year segments, with the trends over each segment shown as dots in Fig. 2a (27 models for a total of 243 dots (Methods)). The regression coefficient of the GSAT trend against the ETP trend and its uncertainty of 5–95% range, based on 5,000 bootstrap samples with replacement for each model, is shown in the inset plot. **b**, Scatter plot as in **a**, but the dots are based on the 53-year period of 1850–1902 from historical simulations. The regression coefficient shown in the inset plot is based on bootstrapping one random ensemble member per model. **c**, Histograms and fitted Gaussian PDFs of the simulated raw GSAT trend (blue) and GSAT trend with ETP-congruent internal variability removed by regression (red), with the GSAT trend calculated from 1970 to 2022. The vertical dashed lines show raw GSAT trends from observations. The observed GSAT trends with ETP-congruent variability regressed out are shown in the black histogram.

**Extended Data Fig. 1:**
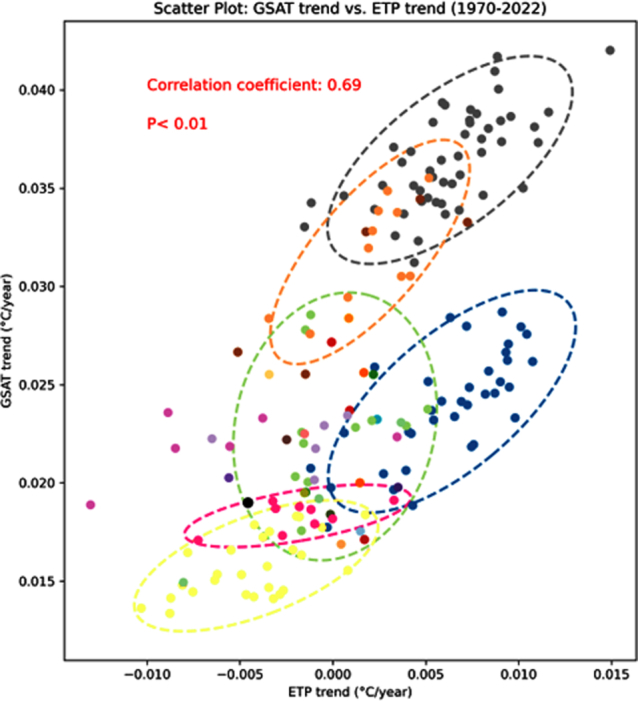
Influence of unforced eastern tropical Pacific internal variability. Similar to Fig. 2a,b but over the period of 1970-2022. Note that inter-model variations in the forced response of the ETP trend have been regressed out; see Methods. The ellipses represenet 5-95% CI for models with more than 6 ensembles. The black dot represents observations. The dots in various colors represent different models in use, and the model names can be referenced in Fig. 1.

As shown in Fig. 2a,b (Fig. 2a is based on pre-industrial control simulations, whereas Fig. 2b is based on the quasi-pre-industrial period 1850–1902 in the historical simulations, over which radiative forcing was relatively constant), the variability of the GSAT trend is well correlated with the variability of the ETP trend (*r* = 0.67, *P* < 0.001 in the pre-industrial control, *r* = 0.66, *P* < 0.001 over the 1850–1902 period and *r* = 0.69, *P* < 0.001 over the 1970–2022 period; Extended Data Fig. 1). This suggests that part of the variance of the GSAT trend can be explained by internal variability in the ETP region. The positive correlation indicates that a warming ETP trend tends to increase the GSAT trend and a cooling ETP trend tends to decrease it, consistent with the physical interpretation of the pattern effect discussed in the literature^17,18,20^. Relative cooling in the ETP where air descends strengthens low-level atmospheric stability. These conditions contribute to the formation of low-level clouds and increase the reflection of solar radiation, which reduces global mean warming. Conversely, relatively positive ETP trends weaken low-level atmospheric stability, which results in a positive cloud radiative feedback leading to a higher global mean warming trend^14,18,20–22^.

Based on the relationship shown in Fig. 2a and confirmed by Fig. 2b, the contribution of unforced ETP internal variability to the GSAT trend can be removed by regression (Methods). The observed 1970–2022 GSAT trend increases with removal of the cooling influence associated with the ETP SST trend, approaching the median of the model simulations (Fig. 2c). In addition, the variance of the simulated GSAT trend is reduced after removing unforced ETP internal variability (comparison of the red PDF and blue PDFs in Fig. 2c).

## Performance of global mean temperature trend as a constraint

**Extended Data Fig. 2:**
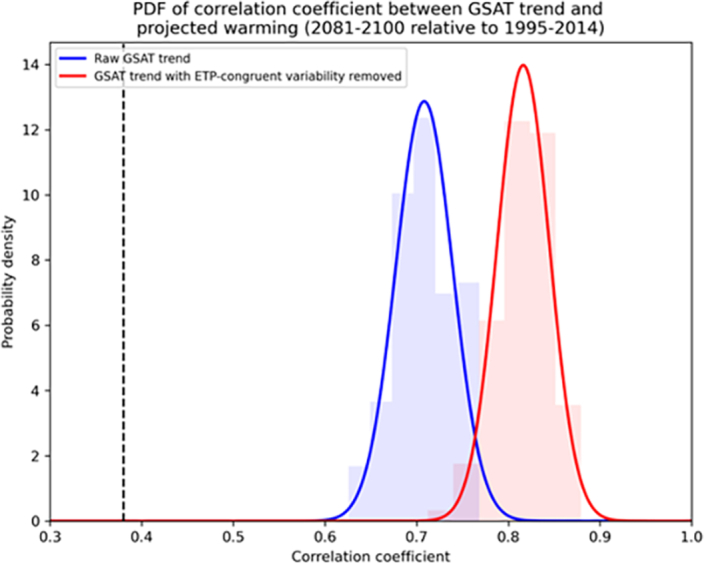
Intermodel correlation between GSAT trend and projected GSAT changes. The correlation coefficients are between GSAT trend (based on 1970-2022) and projected GSAT changes under SSP 5-8.5 (in 2081-2100 relative to 1995-2014). This plot shows histograms and corresponding fitted PDFs of the correlation between the historical GSAT trend and projected GSAT changes across all models. Correlations are based on 5000 random selections of one realization per model. The black dashed line in the plot shows the critical value of correlation coefficient that is significant at the 0.05 level.

Based on an evaluation of trends calculated over a range of periods, the GSAT trend over the period 1970–2022 is optimal for use as an observational constraint on warming. This is supported by its strong inter-model correlation with projected warming and relatively long duration, which reduces the impact of internal variability (Methods). Calculating the GSAT trend over periods with start dates before 1970 reduces the correlation coefficient (*r*), probably because of inter-model differences in aerosol forcing and response over these periods, which are uncorrelated with future warming^26,27^ (Methods). Removing internal variability in the GSAT trend due to unforced ETP variability is hypothesized to enhance the emergent relationship between historical and future GSAT trends. A stronger relationship is expected because this should isolate the forced response in the historical GSAT trend, and the forced response should be well correlated across models with future long-term projected warming. As shown in Extended Data Fig. 2, the GSAT trend over 1970–2022 with ETP internal variability removed has a higher mean correlation with future global mean warming (0.83) than the raw GSAT trend (0.72).

**Fig. 3:**
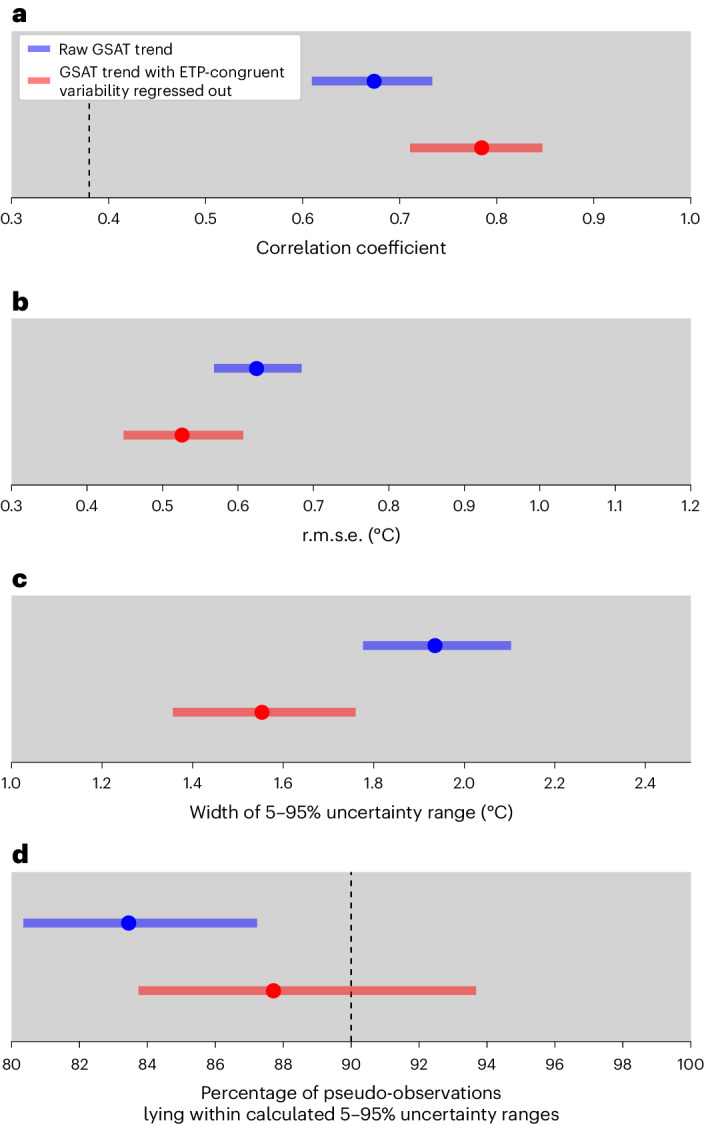
Imperfect model test of constrained GSAT changes. **a**, Correlation coefficients between pseudo-observations and means of constrained GSAT changes. **b**, The r.m.s.e. of the constrained mean relative to pseudo-observations. **c**, The width (95th percentile minus 5th percentile) of the constrained uncertainty range. **d**, An evaluation of the reliability (the percentage of pseudo-observations that lie within the constrained 5–95% ranges (see Methods for details)). The dashed black lines show the critical value of the correlation coefficient that is significant at the 0.05 level.The bars and circles in **a**–**d** show the 5th–95th percentile ranges and means, respectively, of 5,000 random draws of one ensemble member per model. The GSAT changes are in 2081–2100 relative to 1995–2014 under the SSP5-8.5.

To assess if removing ETP-congruent variability from the GSAT trend results in more accurate constrained projections, we apply a cross-validated imperfect model test for the Shared Socioeconomic Pathway 5 version 8.5 (SSP5-8.5). We treat the simulated historical warming trend from each climate model in turn as pseudo-observations and use a regression-based observational constraint approach with output from the remaining climate models to predict future warming in the withheld model (Methods). Consistent with past results, we find that projections constrained using the raw 1970–2022 GSAT trend outperform unconstrained model projections^1,3,28^^,29^, indicated by corresponding significant positive correlations and reductions in the root mean square error (r.m.s.e.) in Fig. 3a,b. In addition, our imperfect model test results also show that projections constrained using the GSAT trend with ETP-congruent variability removed result in more accurate mean projections relative to pseudo-observations (the red solid lines versus blue solid lines; Fig. 3b) and a narrower constrained 5–95% uncertainty range (Fig. 3c) compared with projections obtained by applying the raw GSAT trend as the predictor. Moreover, we find no evidence of overconfidence in our constrained projections, because around 90% of pseudo-observations lie in the constrained 90% uncertainty ranges resulting from both predictors (Fig. 3d). Results for the SSP1-2.6 (Extended Data Fig. 6) are largely consistent with those obtained for the SSP5-8.5, albeit with somewhat lower correlations and reductions in r.m.s.e., which is expected due to the lower signal-to-noise ratio in the SSP1-2.6 projections. The results shown in Extended Data Fig. 6 do show a slight overconfidence in the constrained projections based on this scenario when using either the raw or filtered GSAT trend as a constraint.

## Observationally constrained projections of global warming

**Fig. 4:**
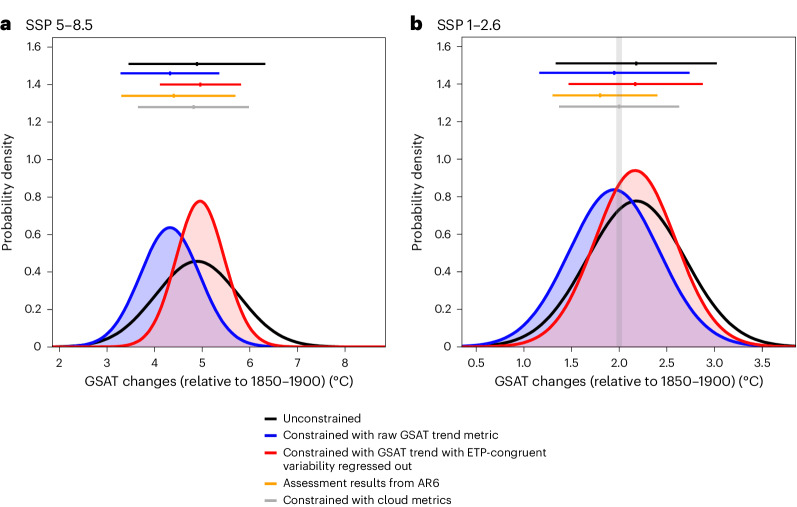
Distributions of constrained and unconstrained GSAT changes. **a**,**b**, GSAT changes are in 2081–2100 (SSP5-8.5 in **a** and SSP1-2.6 in **b**) relative to 1850–1900. The black curves show unconstrained projections, while the blue curves show projections constrained using the raw GSAT trend as a predictor. The red curves show projections constrained using the GSAT trend with ETP internal variability removed as a predictor (the scatter plot illustrating the PDF can be found in Extended Data Fig. 8). The corresponding 5–95% uncertainty ranges are shown by the horizonal lines with means shown as vertical tick marks. The orange lines show IPCC-assessed projections^33,34^. The grey lines show constrained projections from multivariate linear regression models, based on a set of climatological metrics derived from tropical and subtropical low-level cloud. Following Liang et al.^6^, the first of these two cloud metrics is derived from the regression coefficient of monthly marine boundary layer cloud fraction against SST changes across the seasonal cycle averaged over the subtropics. The second cloud metric characterizes cloud shallowness defined by Brient et al.^10^ based on the ratio of cloud fraction at levels below 900 hPa to that below 800 hPa over weakly subsiding tropics based on a climatology over the 1980–2005 period. The faint grey vertical line in **b** marks the 2 °C global warming threshold of the Paris Agreement. Similar results relative to the base period 1995–2014 are shown in Extended Data Fig. 7.

We now apply the observed GSAT trend over 1970–2022 with ETP-congruent variability removed as a predictor to constrain projected global mean warming relative to 1995–2014 (ref. ^29^). Adding observed warming in 1995–2014 relative to 1850–1900 of 0.85 °C (0.69–0.95 °C)^30^, with uncertainties added in quadrature, we can calculate warming relative to 1850–1900 (Fig. 4). For constrained projections under the fossil fuel-intensive high-emissions scenario SSP5-8.5 (refs. ^31,32^), the range constrained by the GSAT trend with the ETP internal variability removed is 4.13–5.82 °C, which is about half as wide as the unconstrained range of 3.45–6.32 °C. Moreover, this constrained range is also substantially narrower than that constrained using the raw GSAT trend (3.30–5.36 °C; Fig. 4a) and the corresponding Intergovernmental Panel on Climate Change (IPCC) constrained range of 3.3–5.7 °C (ref. ^33^). Furthermore, our mean is also notably higher than the IPCC best estimate (Fig. 4a). Note that the IPCC warming projections were based both on projections constrained directly using the observed GSAT trend^1,3,28^ and on an emulator, which was indirectly constrained in part using historical warming. For constrained projections based on a low-emissions scenario in which carbon dioxide emissions are reduced to net zero by the 2070s (SSP1-2.6)^31,32^, the warming range constrained using the GSAT trend with the ETP-congruent variability removed is 1.47–2.88 °C, compared with the unconstrained range of 1.33–3.02 °C, the IPCC assessed range of 1.3–2.4 °C (ref. ^33^) and the range constrained by the raw GSAT trend of 1.15–2.74 °C (Fig. 4b). Whereas the IPCC assessed that warming relative to 1850–1900 was unlikely (*P* < 66%) to exceed 2 °C this century under the SSP1-2.6 scenario^34,35^, our results imply that warming is likely (*P* > 66%) to exceed 2 °C under this scenario (Fig. 4b)^33,34^. Repeating these calculations using the GSAT trend extended to 1970–2023 as predictor, we find there is no substantial difference between the constrained projections results in this case.

The GSAT trend predictor with the unforced ETP variability removed thus results in a higher constrained mean and narrower constrained uncertainty range, compared with projections constrained with the raw GSAT trend (Fig. 4). The projections constrained using the GSAT trend with the unforced ETP variability removed are also in closer agreement with the projections constrained using climatological cloud metrics (Fig. 4), which are only weakly affected by internal variability^6^.

## Discussion

Much of the difference between observed historical GSAT trends and historical GSAT trends in individual climate model simulations is associated with unforced internal variability in the ETP region. While the ETP region covers only 8.2% of the global surface, it can significantly impact low-frequency GSAT variability^17^. This is because the Earth’s energy budget, particularly the feedback related to low-level clouds in the tropical Pacific, is highly sensitive to SSTs in the ETP region. We find large internal variability in the ETP trend and a strong connection between ETP variability and GSAT trend variability in CMIP6 simulations. To produce a more robust emergent constraint, we remove the variability in the GSAT trend congruent with unforced variability in the ETP region.

The observed GSAT trend over the 1970–2022 period with ETP-congruent internal variability removed is close to the CMIP6 multi-model mean trend, in contrast to the raw observed trend, which is well below the CMIP6 multi-model mean trend. Assessing the emergent relationship with an imperfect model test, we obtain more accurate projections with narrower uncertainties by applying the past GSAT trend with ETP-congruent internal variability removed as a constraint. Hence, by appropriately removing internal variability, we can enhance the emergence of the forced signal within the historical predictor. This enables us to better constrain projected warming.

We find a larger constrained projected warming with a narrower uncertainty range using the GSAT trend with ETP-congruent variability removed as a constraint, compared with using the raw GSAT trend. The width of the unconstrained 5–95% uncertainty range of 2081–2100 warming relative to 1850–1900 is reduced by 42% for SSP5-8.5 and by 17% for SSP1-2.6 when constrained using the 1970–2022 GSAT trend with ETP-congruent variability removed, compared with a reduction of only 28% and 6% using the raw 1970–2022 GSAT trend as a constraint. The reduction in uncertainty is larger for SSP5-8.5 than for SSP1-2.6. This is because the forced response and associated uncertainties are larger in SSP5-8.5 than in SSP1-2.6, while internal variability is comparable in the two scenarios. Consequently, there is greater scope for observational constraints to reduce uncertainties in SSP5-8.5 compared with SSP1-2.6. The constrained projection range using the GSAT trend with ETP-congruent variability removed is more consistent with results obtained using climatological cloud metrics as a constraint^6^, which may be because both constraints have smaller contributions from internal variability. Nonetheless, the method we present here outperforms the cloud constrained projections, with narrower uncertainties for SSP5-8.5. Our best-estimate observationally constrained warming projections are very close to the CMIP6 multi-model means for both SSP5-8.5 and SSP1-2.6. These results thus indicate that, on average, the CMIP6 models do not warm too strongly, in contrast to other suggestions in literature^1,36^.

As in other studies applying emergent constraints, this study assumes exchangeability between models’ simulations and the real world^28^, including a realistic representation of the relationship between tropical Pacific SSTs and GSAT. If the CMIP6 models systematically misrepresent the relationship between tropical Pacific SST and GSAT trends, this is likely to bias the constrained GSAT projections. Further critical examination of this exchangeability assumption is an important direction to advance the study. The presence of a systematic bias in tropical Pacific SST trends in models, for example, due to misrepresented Antarctic meltwater^37^ or due to misrepresented aerosol forcing, might imply that the muted ETP warming would persist into the future, reducing the accuracy and reliability of our approach. However, we note that the IPCC^38^ recently assessed that internal variability was a major driver of the muted warming relative to the forced response in the ETP over recent decades, which supports our approach.

Our results imply that future climate warming inferred directly from the raw past warming trend^28,33^ will be biased low due to the influence of unforced internal variability. Whereas the IPCC assessed that warming is unlikely to exceed 2 °C this century under a low-emissions scenario with ambitious emissions reductions (SSP1-2.6), based on our results, 2 °C warming is likely to be exceeded under this scenario. This fact further emphasizes the need for rapid and deep emissions reductions to meet the Paris Agreement goal of limiting global warming to well below 2 °C. In addition, uncertainties in climate projections are key to planning adaptation, and many climate variables and impacts scale with the GSAT changes^39^. Therefore, the substantial narrowing of uncertainties in projections that we demonstrate will have substantial benefits for adaptation planning^33,40,41^.

## Methods

### Model data and observation

This study uses simulations of GSAT change in 2081–2100 relative to 1995–2014, from CMIP6 model simulations driven by both SSP5-8.5 and SSP1-2.6 scenarios^16,43^. We use the historical GSAT trend over the period 1970–2022 as a predictor. The historical period 1970–2022 is used for calculating the past warming trend because the correlation with projected warming is highest for trends calculated over this period (outlined in ‘The optimal time period of past trends for constraint’). This is probably because the GSAT trend response to aerosol changes over this period is relatively small compared with other longer periods so that the dominant external forcing is greenhouse gases, as in the future^3^. In addition, the 53-year 1970–2022 period reduces the influence of decadal to multi-decadal internal variability relative to the use of shorter periods. The CMIP6 model simulations used to calculate the GSAT trend (based on near-surface air temperature) and ETP trend (based on SST) are listed in Supplementary Table 1. The corresponding observed GSAT trends are derived from HadCRUT5^44^, Berkeley Earth^45^ and NOAAGlobalTempv5^46^, while observed ETP trends are derived from HadCRUT5^44^ and Extended Reconstructed Sea Surface Temperature version 5 (ERSSTv5)^47^.

### Estimating the impact of ETP variability on the GSAT trend

We construct a linear regression model of the relationship between the GSAT trend and ETP trend using pre-industrial control simulations from each climate model. We extract the last 477 years of the control simulation of each model and divide this into nine 53-year segments. We calculate a regression coefficient using nine ensemble member per model and bootstrap with replacement 5,000 times to calculate uncertainties (Fig. 2a). We also check our results by carrying out a similar analysis using a 53-year period from the historical simulation with minimal trends in radiative forcing (1850–1902).

### Removing ETP-congruent variability in the GSAT trend

**Extended Data Fig. 3:**
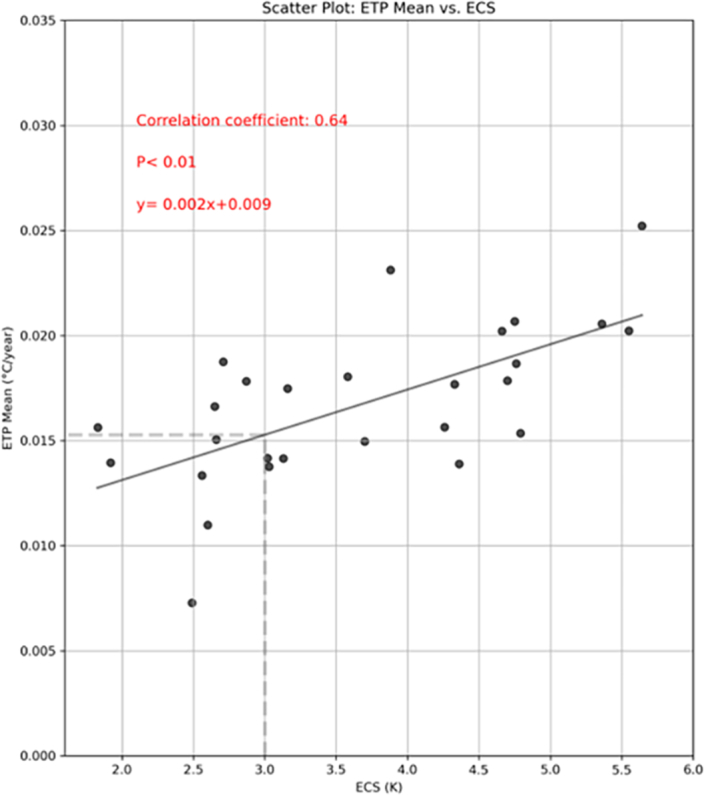
Relationship between ETP trend and ECS. Scatter plot showing ensemble mean of 1970-2022 ETP trend versus ECS for each model, and the corresponding regression fit (gray line). Correlation coefficient between ECS and the ETP trend across the multi-model ensemble and its corresponding *p*-value are shown. The best estimate of forced response in observed ETP trend are shown in the horizontal dashed line, derived from the regression and best estimate of real-world ECS (vertical dashed line).

We use two steps to remove the internal variability in the GSAT trend that is congruent with variations in ETP SST trends. The first step is to estimate unforced internal variability over the ETP region for the set of model realizations. As shown in Extended Data Fig. 3, the forced component of the ETP trend, estimated as the ensemble mean for each model, is moderately correlated with the equilibrium climate sensitivity (ECS) across CMIP6 models (*r* = 0.64, *P* < 0.01; Extended Data Fig. 3). This connection indicates that part of the multi-model variance of the ETP trend can be explained by model differences in forced response.

In particular, a regression model based on the individual models’ ensemble means of ETP trend from historical simulations (denoted as $\bar{{\bf{A}}{\boldsymbol{}}\,}$, with the overbar representing averaging over initial condition ensembles for an individual model) and modelled ECS (${\bf{B}}$) can be established (equation (1)), where ${\alpha }_{1}$ is the intercept and ${{\bf{\upbeta }}}_{{\boldsymbol{1}}}$ is the regression slope; ${{\boldsymbol{\varepsilon }}}_{{\boldsymbol{1}}}$ represents the regression residuals. The number of rows in $\bar{{\bf{A}}}$ and ${\bf{B}}$ is equal to the number *M* of climate models used.

1 $$\bar{{\bf{A}}}={\alpha }_{1}+{{\bf{\upbeta }}}_{{\bf{1}}}{\bf{B}}+{{\bf{\varepsilon }}}_{{\bf{1}}}.$$

The unforced ETP trend (${{\bf{A}}}^{{\boldsymbol{(}}{\boldsymbol{1}}{\boldsymbol{)}}}$; shown on the *x* axis of Extended Data Fig. 1) over 1970–2022 from the historical experiment is estimated by subtracting the forced ETP trend for each model $\bar{{\bf{A}}}$ from ETP trends of individual realizations from that for model ${\bf{A}}$ thus

2 $${{\bf{A}}}^{({\bf{1}})}={\bf{A}}-{\alpha }_{1}-{{\bf{\upbeta }}}_{{\bf{1}}}{\bf{B}}.$$

In the second step, we remove the unforced ETP-congruent internal variability from the GSAT trend in the observations, as well as in each model simulation. We assume that the impact of ETP variability on the GSAT trend in the multi-model CMIP6 ensemble is exchangeable with the real world. This removal is based on the regression relationship established using the pre-industrial control simulations (Fig. 2).

Specifically, the regression coefficient, ${{\bf{\upbeta }}}_{{\boldsymbol{2}}}$, of unforced GSAT trend variations (${\bf{C}}$) against unforced ETP trend variations (${{\bf{A}}}^{{\boldsymbol{(}}{\boldsymbol{2}}{\boldsymbol{)}}}$) is estimated from the pre-industrial control based on regression equation (3), where ${{\boldsymbol{\varepsilon }}}_{{\boldsymbol{2}}}$ represents the regression residuals, as

3 $${\bf{C}}={{{\bf{\upbeta }}}_{{\bf{2}}}{\bf{A}}}^{({\bf{2}})}+{{\boldsymbol{\varepsilon }}}_{{\bf{2}}}.$$

The number of rows in ${\bf{C}}$ and ${{\bf{A}}}^{{\boldsymbol{(}}{\boldsymbol{2}}{\boldsymbol{)}}}$ is equal to *M* times nine (as the control integrations are separated into nine sections). Then, for the historical GSAT trend in 1970–2022, the unforced ETP-congruent variability in GSAT trend (${{\bf{C}}}^{{\boldsymbol{(}}{\boldsymbol{1}}{\boldsymbol{)}}}$) can be estimated by equation (4) as

4 $${{\bf{C}}}^{({\bf{1}})}={{{\bf{\upbeta }}}_{{\bf{2}}}{\bf{A}}}^{({\bf{1}})}.$$

Finally, the historical GSAT trend over the period 1970–2022 (***x***) is used with the ETP-congruent variability (${{\bf{C}}}^{{\boldsymbol{(}}{\mathbf{1}}{\boldsymbol{)}}}$) to estimate the GSAT trend without ETP-congruent variability (${{\bf{x}}}^{{{{\prime} }}}$) as

5 $${{\bf{x}}}^{{\prime} }={\bf{x}}-{{\bf{C}}}^{({\bf{1}})}.$$

We estimate the observed unforced ETP trend (the ERSSTv5 and HadCRUT5 are two observation datasets used to calculate the ETP trend) based on equations (1) and (2) by sampling from a Gaussian distribution fitted to the assessed very likely range of ECS (with a mean equal to the AR6 best estimate 3 °C and a standard deviation of 0.6°C). A range of observed GSAT trends with unforced ETP-congruent variability removed is then estimated using equations (4) and (5). We account for observational uncertainty by sampling the GSAT trend from three datasets (HadCRUT5, Berkeley Earth and NOAAGlobalTempv5) and sampling the ETP trend from two observed ETP datasets (HadCRUT5^44^ and ERSSTv5^47^). The sampling process described here is coordinated with sampling strategies addressing internal variability in observational constrained projections (outlined below).

### Regression model for observational constraint

The constrained projections are obtained using linear regression as described below. We first apply ordinary least-squares regression to fit the linear model ${\bf{y}}=\alpha +{{\bf{x}}}^{{\intercal}}{\bf{\upbeta }}$. The historical predictor is denoted by ${\bf{x}}$ and future predictand is represented by *y*. The number of rows in ${\bf{x}}$ and *y* is equal to the number *M* of climate models used. By introducing an observational estimate ${{\bf{x}}}_{{\bf{0}}}$ of the predictor into the linear regression model, the best estimate of projected warming due to the constraint is denoted by ${\hat{y}}_{0}$. Assuming Gaussian regression errors, the constrained projection has the PDF^48–50^

6 $$p\left(\left.y\right|{{\bf{x}}}_{{\bf{0}}}\right)=\frac{1}{\sqrt{{2\pi \sigma }_{{\hat{y}}_{0}}^{\,2}}}\exp \left(-\frac{{\left(y-{\hat{y}}_{0}\right)}^{2}}{2\,{\sigma }_{{\hat{y}}_{0}}^{2}}\right),$$

where

7 $${\sigma }_{{\hat{y}}_{0}}^{2}{=s}^{2}\left(1+{{\bf{x}}}_{{\bf{0}}}^{{\intercal}}{({{\bf{x}}}^{{\intercal}}{\bf{x}})}^{-1}{{\bf{x}}}_{{\bf{0}}}\right)$$

and

8 $${s}^{2}=\frac{1}{M-2}\mathop{\sum }\limits_{m=1}^{M}{\left(\;{y}_{m}-{\hat{y}}_{m}\right)}^{2}.$$

### Sampling strategies to account for internal variability

**Extended Data Fig. 4:**
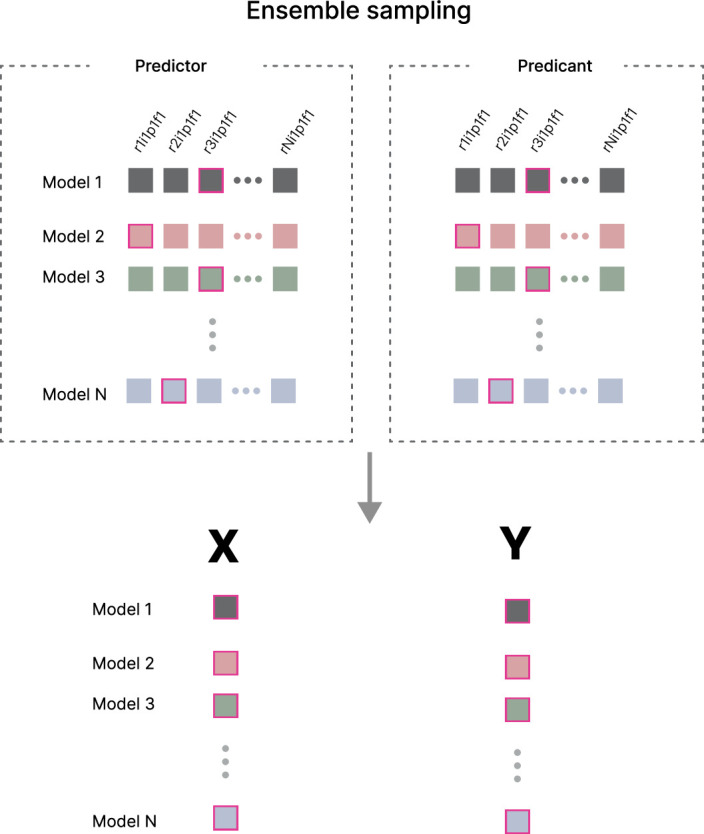
Schematic plot of the constraint sampling procedure. As described in Methods, we randomly sample one ensemble member per model to estimate emergent constraint (repeating this process multiple times to sample uncertainty).

To account for the influence of internal variability, we derive our constrained projections by randomly selecting one ensemble member from each model (see Extended Data Fig. 4 for the schematic plot). We repeat this random selection process 5,000 times during the imperfect model test evaluation and observational constraint procedure. This approach serves two purposes: it mitigates the influence of substantial variations in the ensemble size across different models and enables us to estimate the impact of internal variability in the model simulations on our constrained projections. Additionally, we conduct a sensitivity test to evaluate whether our constrained results are biased by the limited number of models with ensemble sizes larger than one. To account for the contribution of observational uncertainty to the constrained uncertainty, we randomly sample over GSAT and ETP SST observational datasets while sampling over internal variability.

### Imperfect model test

**Extended Data Fig. 5:**
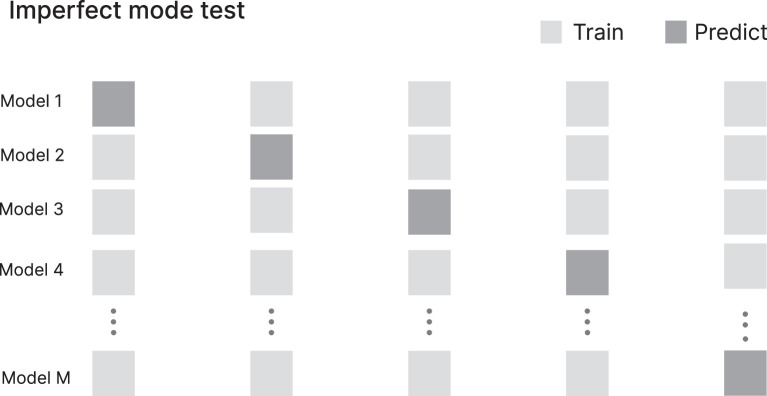
Schematic plot of the imperfect model test. As described in Methods, each model will serve as pseudo-observation to be predicted in turn, and the rest of models can apply emergent constraint method (as the training dataset) to predict the pseudo-observation.

**Extended Data Fig. 6:**
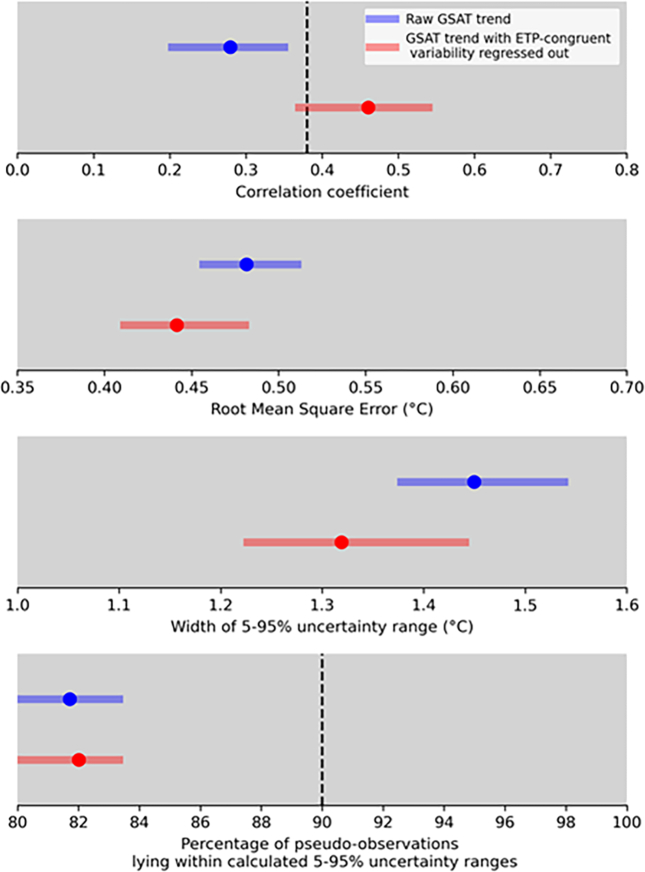
Imperfect model test of constrained GSAT changes. Similar to Fig. 3, but based on SSP1-2.6.

To assess the constraint’s performance we apply a cross-validated imperfect model test^3,6^, in which simulations from each model serve in turn as pseudo-observations and are used to constrain projections by all other models (see Extended Data Fig. 5 for the schematic plot). The evaluation is based on values of the r.m.s.e. and correlation coefficient, both calculated using the pseudo-observations and the means of the constrained imperfect model ensemble. We examine what fraction of pseudo‐observations lie within the 5–95% constrained uncertainty range for each projection, across all models, to assess the coverage probability^3,6^. Ideally, 90% of the pseudo‐observations would lie in the constrained 5–95% uncertainty range in this test. To ensure a fair assessment of predictor performance comparable with observations, instead of using the known ECS for each model to calculate the forced component of its ETP trend, we instead sample ECS from a Gaussian distribution centred on the ECS of each model, with a standard deviation taken from the AR6-assessed likely ECS range. This allows for the generation of a pseudo-observed GSAT trend with ETP-congruent variability removed with appropriate uncertainties.

### The optimal time period of past trends for constraint

**Extended Data Fig. 9:**
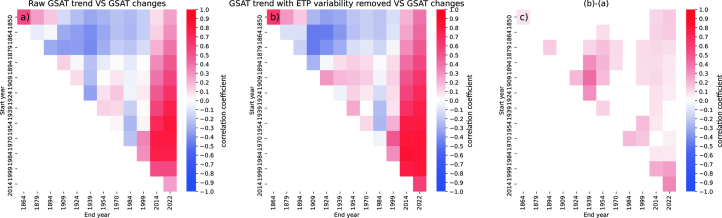
Optimal period for calculating intermodel correlation. The correlation coefficient between the historical GSAT trend (calculated with different start and end years) and future projected warming (between 1995-2014 and 2081-2100 under SSP5-8.5), derived from one ensemble per model. The *y*-axis represents the start years while the *x*-axis represents the end years. a, Correlation coefficient between raw GSAT trend and projected warming. b, Correlation coefficient between GSAT trend with ETP-congruent variability removed. c, Difference between a and b.

To determine the optimal time period to minimize the influence of internal variability and maximize the strength of the statistical relationship between past trends and future warming in the CMIP6 models, we vary the date range over which trends are calculated. We calculate the *r* between historical trends (based on different start and end years) and projected warming between 1995–2014 and 2081–2100 under SSP5-8.5 across CMIP6 models. In this calculation, we consider the first ensemble member of each model. The corresponding results are shown in Extended Data Fig. 9.

As shown in Extended Data Fig. 9a, holding the end year close to the present (for example 2014 or 2022) and making the start year later than the 1970s results in progressively weaker correlation coefficients, due to the increasing influence of internal variability. Probably as the uncertain GSAT response to aerosols is relatively constant over this period^2,3^, the GSAT trend from the 1970s to the present is better correlated with future projected warming compared with periods with earlier start dates (for example, 1850–2022). As shown in Extended Data Fig. 9b,c, by reducing the internal variability, removing the ETP-congruent part of the GSAT trend results in a stronger correlation coefficient with projected warming, especially for GSAT trends calculated over a relatively short period (for example, 1999–2014, 1999–2022 and 2014–2022).

The periods over which the GSAT trend with ETP variability removed exhibits the highest correlation with projected warming are 1970–2022 (with *r* = 0.82), 1984–2014 (*r* = 0.83), 1984–2022 (*r* = 0.81) and 1999–2022 (*r* = 0.81). These periods all show a strong correlation between past trends and future changes across models. Considering that a longer period is expected to reduce the influence of internal variability (and we note that results shown in Extended Data Fig. 9 are based on a single ensemble member), we, therefore, choose 1970–2022 as the best period to constrain future warming.

### Justifying PDF averaging for final constrained uncertainty

As described above, our study samples over initial condition ensembles from climate models, using one ensemble member per model. Each sample gives us a distribution of projected GSAT change (*y* in equation (9)), with a particular mean (*μ*) and standard deviation (*σ*). Hence, multiplying the joint distribution of these two statistics *f*(*μ, σ*), by a conditional distribution *f*(*y* | *μ, σ*), and then integrating over *μ* and *σ* gives us a population estimate *p*(*y*) of the marginal PDF of projected GSAT changes

9 $$p\left(\;y\right)=\iint f\left(\left.y\right|\mu ,\sigma \right)f\left(\;\mu ,\sigma \right){\rm{d}}\mu {\rm{d}}\sigma.$$

Based on our sampling strategy, each derived PDF with its corresponding value of *μ* and *σ* is equally probable. Sampling *μ* and *σ* from their joint distribution and then averaging the resulting conditional distributions hence gives a sample estimate of this population mean distribution of $y$.

### Synthetic test on models’ unequal ensemble sizes

To assess whether the sampling of one random ensemble member per model from models with unequal ensemble sizes biases the constrained distributions, given the availability of only a single ensemble member from many CMIP6 models, we carry out a synthetic data experiment. The experiment utilizes two sets of synthetic data. We create the first set of data by generating synthetic 50-member ensembles for both the historical predictor and future predictand of each climate model. This process assumes Gaussian distributions for all members of the multi-model ensemble. The Gaussian distributions are centred around the ensemble means of individual models, with the standard deviations obtained from CanESM5. The second dataset is generated using the same approach as the first one, but for each model, the ensemble size corresponds to the actual ensemble size, as listed in Supplementary Table 1. We then compare the constrained PDFs derived from each set of synthetic data using our sampling strategy. The procedure is described in more detail below:

Step 1: construct 50-member synthetic ensembles for observable metrics and projected warming for each model. These ensembles are centred on the respective model means and are sampled from Gaussian distributions with the standard deviations matching those of the CanESM5 large ensemble.

**Extended Data Fig. 10:**
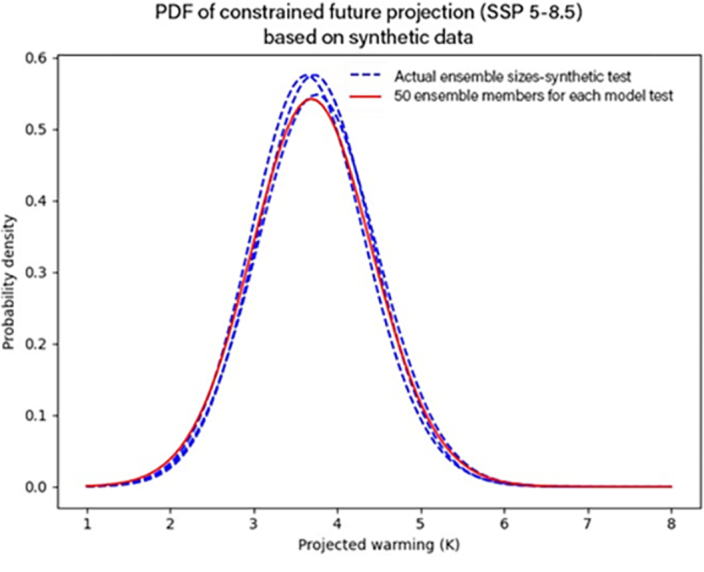
Constrained warming PDFs based on synthetic data experiment. Constrained warming PDFs for global mean surface air temperature change (2081-2100 relative to 1995-2014 under SSP 5-8.5). The red PDF is obtained using the method described in the main manuscript, sampling from synthetic 50-member ensembles for each model. The blue PDFs are derived from the same data but involve first restricting ensemble sizes for each model to match the number of simulations available. Three blue lines are generated through three times of random sampling, for using different subset of ensemble members.

Step 2: randomly select one ensemble member from each of these synthetic 50-member ensembles to generate a PDF of projected warming. Repeat this process 5,000 times, applying the same sampling methodology as described above. The resulting averaged PDF is depicted as the red solid curve in Extended Data Fig. 10.

Step 3: from the 50-member ensembles, select the same number of ensemble members for each model as are actually available, as indicated in Supplementary Table 1. Employ the same sampling method as in step 2 to randomly sample individual ensemble members from this subset and generate an average PDF.

Step 4: repeat step 3 using a different subset of ensemble members. The results are shown with the dashed blue lines in Extended Data Fig. 10.

Our results in Extended Data Fig. 10 show that the distributions obtained using our sampling strategy (dashed blue lines) are similar to the distribution obtained using all available data (red line), suggesting that the limited ensemble sizes available do not have much effect on our resulting PDFs of projected warming.

## Online content

Any methods, additional references, Nature Portfolio reporting summaries, source data, extended data, supplementary information, acknowledgements, peer review information; details of author contributions and competing interests; and statements of data and code availability are available at 10.1038/s41558-024-02017-y.

## Supplementary information

Supplementary Information Supplementary Table 1.

## Figures and Tables

**Extended Data Fig. 7:**
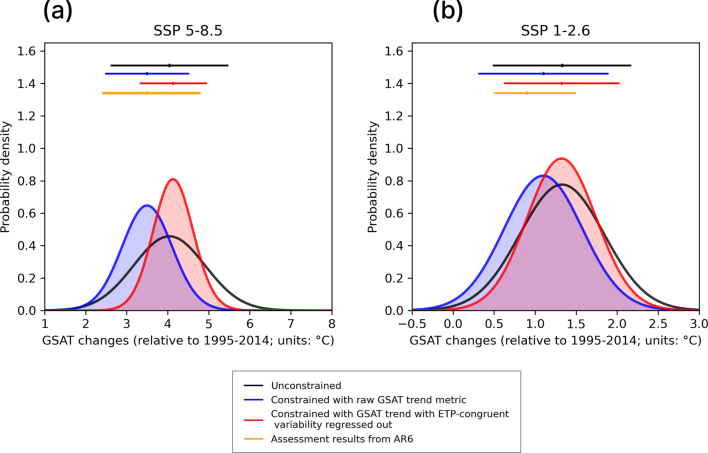
Distributions of constrained and unconstrained GSAT changes. **a**,**b**, Similar to Fig. 4a,b, but the GSAT changes are relative to 1995-2014.

**Extended Data Fig. 8:**
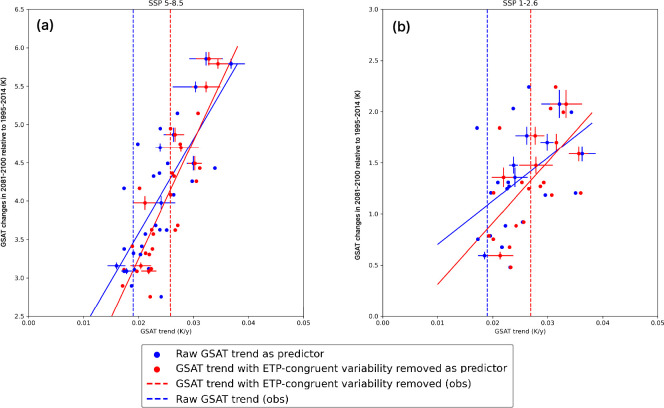
Scatter plot illustrating observational constrained projections. **a**, SSP5-8.5. **b**, SSP1-2.6. The raw GSAT trend metric (in blue) and the GSAT trend with ETP congruent variability removed (in red) are shown on the x-axis whereas the y-axis shows raw projected 2081-2100 warming relative to 1995-2014 from individual models. Each dot with an error bar represents the ensemble mean and standard deviation for one CMIP6 model, with observations indicated by the vertical line. The regression line is fitted by one random ensemble of each model. The constrained PDF, as in Fig. 4, is generated assuming Gaussian distribution of regression errors (see Method). We repeat this process 5000 times using one random ensemble per model to create the PDFs in Fig. 4 and Extended Data Fig. 7.

## Data Availability

We use the CMIP6 simulation data available on https://esgf-node.llnl.gov/projects/cmip6/. HadCRUT5 data are available on https://www.metoffice.gov.uk/hadobs/hadcrut5/. ERSSTv5 data are available on https://psl.noaa.gov/data/gridded/data.noaa.ersst.v5.html.

## Code availability

The computer code necessary to reproduce the main results is provided at Zenodo via 10.5281/zenodo.10999319 (ref. ^51^).
