# Supplementary material for: Accounting for Pacific climate variability increases projected global warming
Source: Nat Clim Chang. 2024 Jun 5;14(6):608–14. doi: 10.1038/s41558-024-02017-y (PMC11636978; doi:10.1038/s41558-024-02017-y)
Supplement: Supplementary file 1 — Supplementary Table 1. [file 41558_2024_2017_MOESM1_ESM.pdf]

nature climate change

Supplementary information https://doi.org/10.1038/s41558-024-02017-y

Accounting for Pacific climate variability increases projected global warming

In the format provided by the authors and unedited

Table of Contents

Table 1. List of CMIP6 Historical, SSP5-8.5, SSP1-2.6 and preindustrial Control simulations used in this study.

Table 1 List of CMIP6 Historical, SSP5-8.5, SSP1-2.6 and preindustrial Control. The numbers of ensemble members used for each experiment are listed in the second through fifth columns. We use all simulations for which the necessary model output is available.

Model name	Historical	SSP5-8.5	SSP1-2.6	preindustrial Control
ACCESS-ESM1	10	10	10	1
AWI-CM-1-1-MR	1	1	1	1
BCC-CSM2-MR	1	1	1	1
CanESM5	50	50	50	1
CESM2	6	2	2	1
CMCC-ESM2	1	1	1	1
CNRM-CM6-1	6	6	6	1
CNRM-ESM2-1	5	5	5	1
EC-Earth3	1	1	1	1
EC-Earth3-CC	1	1	1	1
EC-Earth3-Veg	1	1	1	1
FGOALS-g3	1	1	1	1
GFDL-ESM4	1	1	1	1
GISS-E2-1-G	13	13	13	1
HadGEM3-GC31-LL	4	4	4	1
INM-CM4-8	1	1	1	1
INM-CM5-0	1	1	1	1
IPSL-CM6A-LR	7	7	7	1
MCM-UA-1-0	1	1	1	1
MIROC-ES2L	7	7	7	1
MIROC6	50	50	50	1
MRI-ESM2-0	6	6	6	1
MPI-ESM1-2-LR	9	9	9	1
NESM3	4	4	4	1
NorESM2-LM	3	1	1	1
TaiESM1	1	1	1	1
UKESM1-0-LL	8	8	4	1
